# PlantGF: an analysis and annotation platform for plant gene families

**DOI:** 10.1093/database/baab088

**Published:** 2022-01-17

**Authors:** Jiaxuan Li, Shuai Yang, Xiaojie Yang, Hui Wu, Heng Tang, Long Yang

**Affiliations:** College of Information Science and Engineering and Agricultural Big-Data Research Center, Shandong Agricultural University, Daizong Road No.61, Taian 271018, China; Seed Engineering Technology Center, YuXi ZhongYan Tobacco Seed Co., LTD, Nanxiang Road No.14, Yuxi 653100, China; Agricultural Big-Data Research Center and College of Plant Protection, Shandong Agricultural University, Daizong Road No.61, Taian 271018, China; Agricultural Big-Data Research Center and College of Plant Protection, Shandong Agricultural University, Daizong Road No.61, Taian 271018, China; Agricultural Big-Data Research Center and College of Plant Protection, Shandong Agricultural University, Daizong Road No.61, Taian 271018, China; Agricultural Big-Data Research Center and College of Plant Protection, Shandong Agricultural University, Daizong Road No.61, Taian 271018, China

## Abstract

Gene families contain genes that come from the same ancestor and have similar sequences and structures. They perform certain specific functions within and among different species. Currently, there is no complete process or platform for the rapid analysis of plant gene families. In this study, a comprehensive query and analysis platform of plant gene families, the Plant Gene Family Platform (PlantGF), was constructed. The platform is composed of four main parts: Search, Tools, Statistics and Auxiliary. A total of 2 909 580 gene family members were identified from 138 plant species in PlantGF. The data can be queried in the Search section through a user-friendly interface. A general process for gene family analysis, having nine steps, is provided. The platform also includes four online tools (HMM-Search, BLAST, MAFFT and HMMER) in the Tools section for useful additional analyses. The statistical analysis of the relevant gene families is shown on the Statistics page. Auxiliary pages are provided for data downloading. The datasets for all 138 plant species’ protein sequences and their gene families can be acquired on the Download page. A user’s manual and some useful links are displayed on the Manual and Links pages, respectively. To the best of our knowledge, PlantGF is the first comprehensive platform for studying plant gene families, and it will make important contributions to plant gene family-related research.

**Database URL**: http://biodb.sdau.edu.cn/PGF/index.html

## Key points

- Genome-level annotated protein sequences from 138 plant species have been used and analyzed in this research.
- We searched all potential families and domains from the 138 species.
- A total of seven useful online bioinformatic tools have been provided for the diverse analysis of the families.
- Complete data are provided for users to start their own comprehensive analysis.
- Based on all the above data, a web-friendly database has been constructed for all the researchers even without any family basic knowledge to make a rapid and effective analysis.

## Introduction

A gene family is formed through the duplication and mutation of the same ancestor. Additionally, family members are defined as containing the same domains. For instance, WRKY genes, which all contain the W-R-K-Y domain, are important components of plant defense response-related signal transduction (1). Generally, these domains have conserved sequences that easily form stable three-dimensional structures, which then determine their particular function.

With improved sequencing technologies, the genome-wide sequencing of a series of important plant species has been completed, promoting research on plant genomics at the molecular level. Evolutionary biologists are now exploring the evolutionary laws of genomes using whole-genome data. To date, more than 500 plant species have been sequenced and released on public platforms (2). Currently, a huge number of gene families in one or more plants have been studied, but most of this research has been focused on families involved in specific plant characteristics, such as the SWEET family in pineapple (3), which is involved in the sugar transport process, the PPR family in tomato (4), which is involved in growth and development (4), and the WRKY family in tobacco (5), which is involved in stress resistance processes. However, there is no comprehensive platform to display and analyze all the available gene families of a plant species. This prompted the construction of a gene family database that would provide convenient access to data for all plant gene families.

Two comprehensive databases, Pfam (6) (http://pfam.xfam.org/) and InterPro (7) (http://www.ebi.ac.uk/interpro/), focus on gene family research. The Pfam database is a comprehensive platform for gene family processing, and it is dedicated to collecting specific domains and then identifying conserved domains using a Hidden Markov Model (HMM) algorithm. Interpro provides functional analyses of proteins through classification and domain and important site predictions. However, these databases mainly focus on gene families and their functions. There is currently no complete plant gene family study and self-analysis database. Therefore, it is necessary to establish an omnibus platform of plant gene families. In this study, the plant gene family database was developed to query all the gene families of sequenced plants and their functional annotations, and it provides some analytical tools and useful links. The database is a resource and analysis platform through which plant gene families can be well studied, as well as evolutionary relationships.

## Platform content and web interface

**Figure 1.**
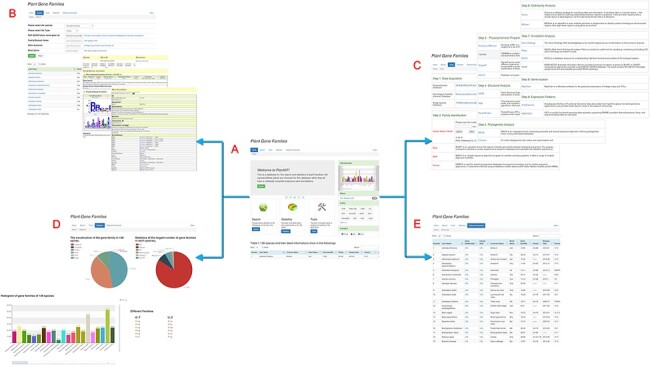
Main PlantGF web page. (A) PlantGF homepage: provides quick entry paths to all main parts. (B) Search: contains 2 909 580 gene family members and their specific detail annotations. (C) Tools: consists of nine steps in gene family analysis. Among that, four online tools (HMM-Search, BLAST, MAFFT and HMMER) also exist. (D) Statistics: statistics among these gene families. (E) Datasets Download: provides download links of 138 plant species’ protein sequences and their gene families.

Plant Gene Family (PlantGF) is a database committed to the gene families of sequenced plants, and it provides family analysis-related online tools. The database contains four main components: Search, Tools, Statistics and Auxiliary. Search and Tools are the two core components of the platform, and there are some necessary related modules (i.e. Manual, About us and Links). The result is an open and user-friendly web interface (Figure 1).

PlantGF’s main components are as follows: (i) Search; this section contains detailed data on 138 plant gene families. These gene families can be queried using species name, family type, species family ID, family name, Pfam accession and Description. In addition, some exhaustive gene annotations, including Pfam (6), Prosite (8), EMBL (9), KEGG (10) and GO (11) are displayed on secondary webpages. (ii) Tools; a series of popular and convenient tools for gene family analyses are shown. There are nine common steps: Data Acquisition, Family Identification, Physicochemical Property Analysis, Structural Analysis, Phylogenetic Analysis, Collinearity Analysis, Annotation Analysis, Gene Location and Expression Patterns. Among them, four specific online tools applicable to this database, HMM (12), BLAST (13), MAFFT (14) and HMMER (12), are marked in red on this page. (iii) Statistics; this section contains the numbers of family members and their specific distributions. (iv) Auxiliary; this section provides the protein sequences and gene families found in 138 plant species. Some useful links and the user’s manual are also available. In addition, gene family types and member numbers in different species are displayed and can be downloaded in file tree form on the Statistics page.

## Gene family

**Figure 2.**
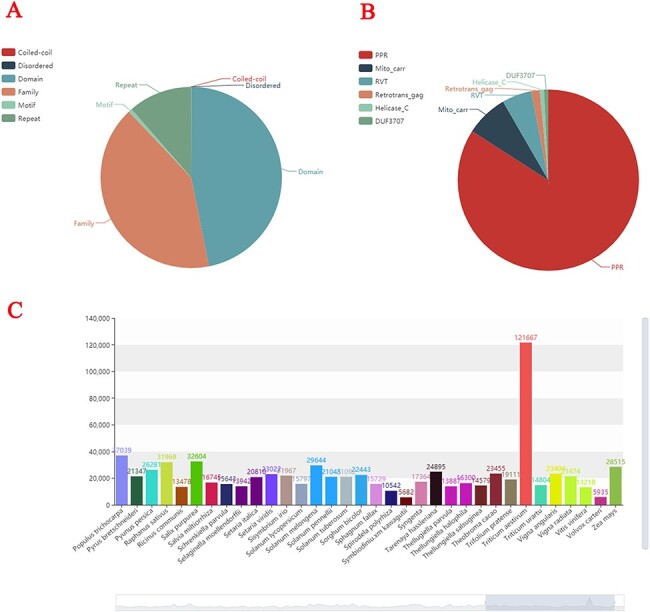
Statistics of plant gene families. (A) The composition structure of 138 plant species’ genes. (B) Statistics of the largest number of gene families in each species. (C) The number of gene families of each species.

All the gene Family, Domain, Coiled-coil, Disordered, Motif and Repeat have been identified in the 138 plant species’ gene sequences attained from public platforms (Figure 2A). In total, 2 909 580 gene family members were identified, and some necessary annotations were developed for each gene. Approximately 80% of plant species contain the PPR gene family, followed by the Mito_carr gene family (Figure 2B). *Triticum aestivum* has the largest number of gene families at 121 667, whereas *Ostreococcus lucimarinus* has the smallest number at 3686 (Figure 2C).

All the gene family datasets and their annotations are stored on the Search page. Users can search these data using checkboxes: Species (138 plant species), Type (Family, Domain, Coiled-coil, Disordered, Motif and Repeat), PGF/short name-gene id (PGF-ID), Family name, Pfam accession and Description. The resulting specified data will appear in a dynamic table after users click ‘Search’ in the current page. The annotation page contains three main parts: Gene sequences, Family/Domain information and Family Gene annotations. (i) Gene sequences; the detailed sequence information and download link are shown. (ii) Family/Domain information; using the annotation data from Pfam (6) and Prosite (8), basic information and a detailed description of the family are provided. In addition, the HMM can be downloaded. (iii) Family Gene annotations; detailed annotations for the queried gene, including SwissProt, EMBL (9), KEGG (10) and GO (11) results are shown. Furthermore, a table of species names, including their short and common names, is provided on the Search page to facilitate user queries.

## Tools

Nine analyses-related steps powered by 26 software programs are shown on the Tools page. The nine steps are Data Acquisition, Family Identification, Physicochemical Property Analysis, Structural Analysis, Phylogenetic Analysis, Collinearity Analysis, Annotation Analysis, Gene Location and Expression Patterns (Table 1).

**Table 1.** Nine steps of gene family analysis

Steps	Name	Tools
1	Data Acquisition	Expression Patterns; Homologous Families (Genera) Database; Single species Database
2	Family Identification	HMM-Search (12); BLAST (13); MAFFT (14); HMMER (12); CDD-NCBI (24); DNAman; MEME (25); SMART (26)
3	Physicochemical Properties	Compute pI/Mw tool (27); TMHMM (28); SingnalP (29); CELLO (30)
4	Structural Analysis	GSDS (31); PDB (32); PredictProtein (33)
5	Phylogenetic Analysis	MEGA (34); Evolview (35)
6	Collinearity Analysis	Cricos (36); MCscanX (37)
7	Annotation Analysis	Gene Ontology (11); Wego (38); KEGG (10); KAAS (39)
8	Gene location	MapChart (40)
9	Expression Patterns	ArrayExpress (41); NCBI-GEO (42)

In addition, four online tools are provided in the Tools section to promote analyses among gene families. HMM-Search provides a simple way to search the HMMs Users just need to input keywords in the textbox and click ‘Submit’. The results will be displayed in a new window. The BLAST software was mainly developed using PlantGF, and the BLAST library consists of 138 plants. Users can input query sequences and select appropriate parameters to inform their results. MAFFT is a multiple sequence alignment program for Unix-like operating systems that can be used for preparing multiple sequence alignment files to develop phylogenetic trees and for HMMER Build. Two main HMMER modules, HMMER Build and HMMER Search, are also provided. HMMs can be developed using HMMER Build and the results of MAFFT. HMMER Search requires users to upload the target HMM and choose plant species to identify their target genes. All the results can be visualized online or can be download to a local computer through the results page.

## Statistics

Statistical analyses are displayed using the different charts available in this section. The construction of a gene family in 138 species and the gene families with the most members in each species are shown in two pie charts. Their detailed information appears when the mouse is passed over the target area. Statistics on gene family types and numbers of different species are shown in the form of tree files. The species are classified using the initials of their scientific names. The number of gene families in each species is shown using a dynamic histogram at the bottom of the page. Users can also turn the mouse wheel to see specific species.

## Materials and methods

### Dataset collection

Identified protein sequences were obtained from species-specific databases and public comprehensive platforms. In total, 13 Brassicaceae family species, including *Aethionema arabicum, Arabidopsis thaliana* and *Brassica napus*, were downloaded from the Brassica Database (http://brassicadb.org/brad/index.php) (15); 12 Solanaceae family species, including *Nicotiana tabacum, Solanum tuberosum* and *Solanum lycopersicum*, were downloaded from the Sol Genomics Network (https://solgenomics.net/) (16); 4 Rosaceae family species, including *Prunus mume, Pyrus bretschneideri* and *Malus × domestica*, were downloaded from Genome Database for Rosaceae (https://www.rosaceae.org/) (17); 16 Grass family species, including *Aegilops tauschii, Hordeum vulgare* and *Oryza sativa*, were downloaded from Gramene (http://gramene.org/) (18) and 3 Cucurbitaceae family species, *Citrullus lanatus, Cucumis melo* and *Cucumis sativus*, were downloaded from Cucurbit Genomics Database (http://cucurbitgenomics.org/) (19). The remaining species came from comprehensive plant databases, such as Phytozome (http://www.phytozome.net) (20), PlantGDB (http://www.plantgdb.org/) (21), NCBI (https://www.ncbi.nlm.nih.gov/), EnsemblPlants (http://plants.ensembl.org/index.html) and PMDbase (http://www.sesame-bioinfo.org/PMDBase/) (22).

### Gene family identity

In this study, Perl script-based Pfamscan software was used to input each protein sequence in a Linux environment with the default parameters. All the data processing and statistics were performed in Perl script, R script and Echarts.

### Gene family annotation

Currently, different annotation databases, like Pfam (6), GO (11), KEGG (10), Uniprot (23) and Prosite (8), contain massive amounts of accurate annotation data, and data can be exchanged among these databases. A one-to-one correspondence for these gene families was annotated using the Pfam accession number of each gene family. Furthermore, it was not possible to obtain accurate annotations for every sequence in every plant. To help the user explore an unknown gene with our platform, we chose genes of well-studied model plants, such as *A. thaliana* and *O. sativa*, as the annotation sources for the gene family. Using this information, researchers may infer the function and origin of their research targets.

### Database implementation

The Python Web framework is popular for constructing databases. First, the detailed annotations of gene families were stored in MySQL, in which data manipulation and maintenance were also performed. Then, uWSGI was used together with HTML and Bootstrap to construct the users’ access interface. Additionally, Flask, BioPython, Perl Scripts, Echarts and Javascript were all required to connect MySQL and uWSGI.

## Conclusions

PlantGF is a comprehensive platform that was developed for the study of plant gene families. It provides 2 909 580 gene family members and their specific detail annotations from 138 plants. Furthermore, the incorporation of several useful tools makes it easy for users unfamiliar with bioinformatics to perform plant family-related scientific research. The platform will be updated continuously as new plant sequences are generated and new bioinformatics tools emerge.
